# Comparing rates of atrioesophageal fistula with contact force-sensing and non-contact force-sensing catheters: analysis of post-market safety surveillance data

**DOI:** 10.1007/s10840-019-00653-5

**Published:** 2019-11-22

**Authors:** Hugh Calkins, Andrea Natale, Tara Gomez, Alex Etlin, Moe Bishara

**Affiliations:** 1 Johns Hopkins Medical Institutions, 600 N. Wolfe Street, Sheikh Zayed Tower 7125R, Baltimore, MD USA; 2 Texas Cardiac Arrhythmia Institute, St. David’s Medical Center, 3000 N. I-35, Suite 720, Austin, TX 78705 USA; 3 Biosense Webster Inc., 33 Technology Dr, Irvine, CA 92618 USA

**Keywords:** Atrial fibrillation (Afib), Atrioesophageal fistula (AEF), Contact force, Radiofrequency (RF) ablation, THERMOCOOL SMARTTOUCH®, CARTO® 3

## Abstract

**Purpose:**

There is limited data on the specific incidence of serious adverse events, such as atrioesophageal fistula (AEF), associated with either contact force (CF) or non-CF ablation catheters. Since the actual number of procedures performed with each type of catheter is unknown, making direct comparisons is difficult. The purpose of this study was to assess the incidence of AEF associated with the use of CF and non-CF catheters. Additionally, we aimed to understand the workflow present in confirmed AEF cases voluntarily provided by physicians.

**Methods:**

The number of AEFs for 2014–2017 associated with each type of catheter was extracted from an ablation device manufacturer’s complaint database. Proprietary device sales data, a proxy for the total number of procedures, were used as the denominator to calculate the incidence rates. Additional survey and workflow data were systematically reviewed.

**Results:**

Both CF and non-CF ablation catheters have comparably low incidence of AEF (0.006 ± 0.003% and 0.005 ± 0.003%, respectively, *p* = 0.69). CF catheters are the catheter of choice for left atrium (LA) procedures which pose the greatest risk for AEF injury. Retrospective analysis of seven AEF cases demonstrated that high power and force and long RF duration were delivered on the posterior wall of the left atrium in all cases.

**Conclusions:**

CF and non-CF ablation catheters were found to have similar AEF incidence, despite CF catheters being the catheter of choice for LA procedures. More investigation is needed to understand the range of parameters which may create risk for AEF.

## 1 Background

During radiofrequency (RF) ablation of cardiac arrhythmias, stable catheter-tissue contact is an important factor for making contiguous transmural lesions and is a strong predictor of 12-month procedural success [1–3]. For that reason, contact force (CF)-sensing catheters are widely used as part of a strategy for the treatment of arrhythmias [4, 5]. Atrial fibrillation ablation procedures involve delivering RF on the posterior wall of the left atrium, often in close proximity to the esophagus. The associated risk of atrioesophageal fistula (AEF) is a rare but potentially lethal consequence of RF ablation [4, 6, 7].

The THERMOCOOL SMARTTOUCH® catheter is a CF ablation catheter that provides real-time CF data in combination with the CARTO® 3 three-dimensional (3D) electroanatomical mapping system. The safety of CF technology has been demonstrated in clinical trials [1–3], though it has been difficult to systematically compare the safety profiles of CF and non-CF catheters. The FDA’s Manufacturer and User Facility Device Experience (MAUDE) has been previously used to estimate the incidence of AEF for each catheter type. However, the MAUDE database does not contain any information about the usage volume of each catheter type. Without this information to serve as a denominator, the incidence will naturally be higher for the catheter type with the bigger volume due to higher opportunity.

**Fig. 1:**
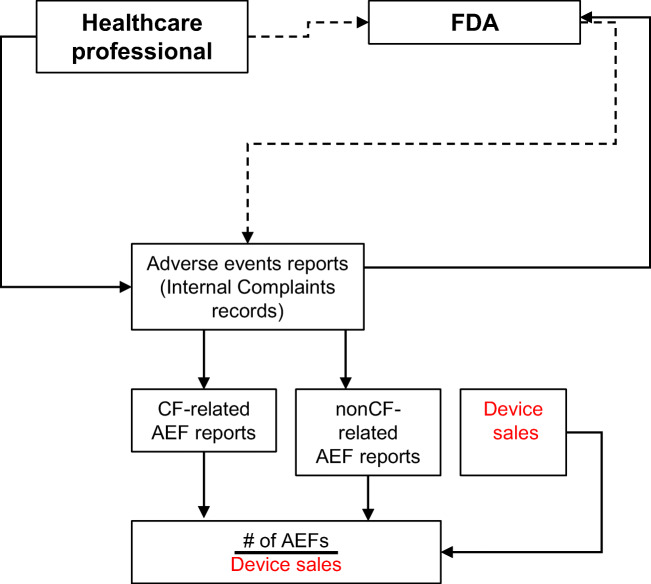
Approach to complaint rate calculations. A healthcare professional or medical device field representative who gains knowledge about an adverse event may report it directly to the manufacturer (solid line). The manufacturer then investigates the complaint and reports to the FDA if it meets reporting criteria set by the FDA. Alternatively, the healthcare professional may decide to report the event directly to the FDA (dashed line), which will relay the complaint to the manufacturer. Thus, for adverse events that meet the FDA criteria of reportability, the MAUDE and Manufacturer databases will contain the same events. In this analysis, the rates of adverse events for each functional family of catheters were normalized to the respective sales volume

Conversely, a manufacturer’s internal complaint database is matched with the FDA database, due to reciprocal notification, i.e., every qualifying adverse event (AEF, etc.) reported to the manufacturer is reported to the FDA and vice versa (Fig. 1). Each report is documented and investigated. This process ensures accurate documentation of the event’s details and prevents incorrect classification of complaints. Therefore, a device manufacturer’s complaint database can be considered as the most complete and accurate repository of complaints/complications associated with its catheters. The device manufacturer’s complaint database has all reports specific to its products including all of those found in MAUDE, and some that might not be available in MAUDE (for instance, a limited number of incidents may not require reporting to the FDA). In addition, the device manufacturer maintains a proprietary record of device sales and performs surveys to estimate device usage. In this analysis, we used the complaint records as the numerator and the proprietary record of device sales (the number of units sold per each catheter type) as the denominator to calculate the true rate of AEF for CF and non-CF catheters (Fig. 1).

The purpose of this study was to assess the incidence of AEF associated with the use of CF and non-CF sensing ablation catheters. Additionally, we aimed to understand the workflow present in confirmed AEF cases voluntarily provided by physicians.

## Methods

### Inclusion criteria

While the THERMOCOOL SMARTTOUCH® catheter was launched in the USA in 2014, it was introduced into the EU in 2011. The device manufacturer’s internal complaints database was queried for all (regardless of procedure type) AEF reports involving the manufacturer’s CF-sensing and non–CF-sensing catheters between January 2010 and December 2017. These dates were selected because the THERMOCOOL SMARTTOUCH® catheter was launched in the USA in 2014. The database is comprised of global complaints of adverse events from physicians, field representatives, and the FDA if a customer reports directly to the FDA. These complaints include late reports of AEFs, as AEFs can occur weeks after the initial procedure. These data were used for temporal trend analysis. As noted above, the period between January 2014 and December 2017 was selected for the main AEF rate analysis to include the year (2014) when THERMOCOOL SMARTTOUCH® catheter was approved by the FDA and therefore tracked within the MAUDE database. All reports of AEF were included in the analysis except cases involving catheters from other device manufacturers, duplicate reports, and reports where catheter type was unknown.

### Complaint rate calculation

The AEF reports queried from the device manufacturer’s complaint database yielded reports involving the following catheters: THERMOCOOL SMARTTOUCH® BIDIRECTIONAL (D-1327-XX), THERMOCOOL SMARTTOUCH® UNIDIRECTIONAL (D-1336-XX), THERMOCOOL SMARTTOUCH® UNIDIRECTIONAL SF (D-1347-XX), and THERMOCOOL SMARTTOUCH® BIDIRECTIONAL SF (D-1348-XX), EZ STEER™ THERMOCOOL™ NAV 4MM (D-1292-XX), EZ STEER® THERMOCOOL™ SF NAV (D-1313-XX and D-1317-XX), NAVISTAR® THERMOCOOL® (D-1197-XX), and THERMOCOOL® SF NAV (D-1315-XX and D-1318-XX) (Biosense Webster Inc., Irvine, CA).

To provide the most accurate estimation of AEF rates and to account for variability between different catheter types, we first calculated the percentage of AEF complaints relative to the sales volume of each type of catheter. Next, we aggregated the data by averaging these rates across the CF and non-CF product lines and reporting a standard deviation.

### Catheter usage surveys

Clinical field employees who attended cases manually logged procedure data into an internal database. These data were extracted to survey what type of catheter, CF or non-CF, was used in LA procedures. This survey data is available only for 2015–2017. To calculate what percent of LA procedures were done with CF vs non-CF catheters, the number of CF or non-CF LA procedures was divided by the total number of recorded LA procedures.

### Statistical analysis

AEF adverse events rates were expressed as an average (± standard deviation (SD)) percentage across CF and non-CF catheter lines. The two-tailed Mann-Whitney *U* test was then used to compare the incidence of AEF between the product lines. A value of *p* < 0.05 was considered statistically significant.

### Retrospective CARTO 3 data analysis

Due to legal and technical constraints, it is difficult to obtain procedure log files for retrospective analysis. Nevertheless, a limited number (7) of anonymized (patient deidentified) CARTO® 3 System log files from procedures involving CF catheters with a documented AEF were voluntarily provided by physicians. Physicians provided anonymized CARTO® 3 System log files from previously completed clinical cases where the patient experienced an AEF between 2014 and 2018. All patient information was deidentified. We only analyzed CF AEF cases because they provide the benefit of assessing CF delivery. We did not make attempts to compare the results with non-CF cases, as this is beyond the scope of the present analysis. Log files were imported into CARTO® 3 System workstation, and individual ablation points on the posterior wall of the left atria were identified using the VISITAG® Module. Average force, maximum power, and duration were recorded and analyzed.

## Results

### Comparison of AEF incidence with CF and non-CF catheters

**Fig. 2:**
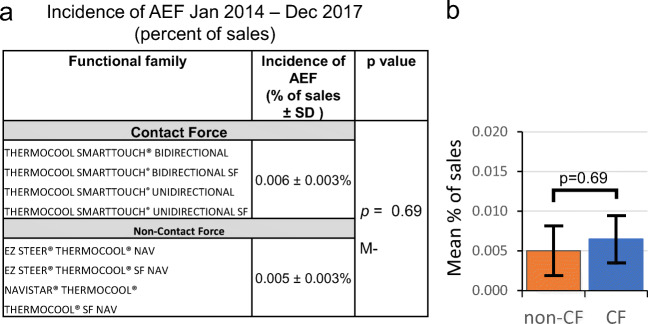
Analysis of complaint and sales data. **a**, **b** Functional families of catheters comprising the CF (blue) and the non-CF (orange) groups in this study and the pooled incidence (percent of sales) of AEF in cases involving these catheters. There was no difference in the AEF rate between the two groups (M-W test, *p* = 0.69). **b** Bar plot summarizing the data in **a**

As shown in Fig. 2a, no difference was observed in the volume normalized rate of AEF between CF and non-CF ablation catheters between 2014 and 2017. The rate of AEF with CF catheters was 0.006 ± 0.003%. This did not differ from the rate of AEF with non-CF catheters (0.005 ± 0.003%, *p* = 0.69).

### Temporal trends in AEF incidence in CF and non-CF catheters

**Fig. 3:**
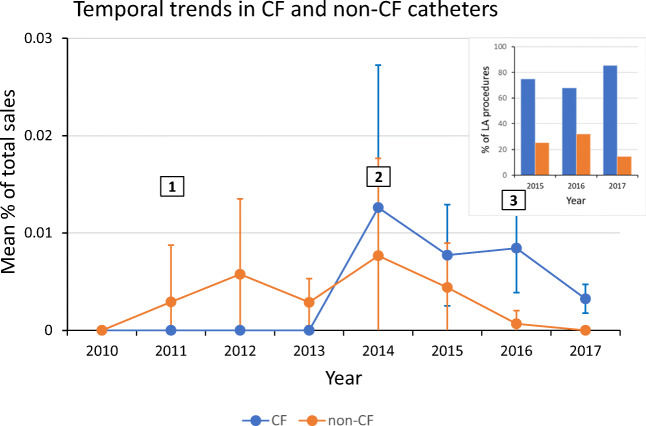
Temporal trends in CF/non-CF AEF incidence and usage. Main panel: Incidence of AEF with CF and non-CF catheters presented over time. Labels indicate the dates of commercial introduction: 1, THERMOCOOL SMARTTOUCH® in the EU; 2, THERMOCOOL SMARTTOUCH® SF in the EU and THERMOCOOL SMARTTOUCH® in the USA; 3, THERMOCOOL SMARTTOUCH® SF in the USA. The trend suggests a greater awareness of AEF coupled with a robust learning curve in the use of these devices. Inset: Rates of usage of CF and non-CF catheters in LA procedures. Since during 2015–2017 CF catheters were used in the vast majority of LA procedures, a direct statistical comparison between the datasets for these years will be inadequate

Figure 3 shows the temporal trends in the incidence of AEF for both CF and non-CF ablation catheters from 2010 to 2017 (Fig. 3, main panel). The dates at which the Smartouch CF catheter was introduced in Europe and the USA are also shown. For both CF and non-CF catheters, the highest incidence of AEF occurred in 2014 and fell thereafter. These trends suggest a greater awareness of AEF coupled with a learning curve in the use of these devices.

To assess the usage of different types of device in different procedures, clinical field representative survey data which collected use data from 2015 to 2017 was analyzed. Because this information has only been collected since 2015, it could not be used for calculating AEF incidence rates over the period used in this report, but it can provide a gross estimate of the types of procedures in which physicians chose to utilize CF and non-CF catheters. In 2015, 2016, and 2017, 75%, 68%, and 85% respectively of LA procedures utilized CF ablation catheters (Fig. 3, inset).

There was no statistical difference in rates between CF and non-CF catheters in any year from 2011 until 2016 (Fig. 3). The CF and non-CF group had non-similar rates of AEF in 2016 and 2017 (*p* value 0.03 and 0.02, respectively). However, as the inset demonstrates, this difference correlates to 2–5-fold more use of CF catheters in LA procedures where the potential for AEF exists. In addition to usage bias, data in 2016 and 2017 are skewed from previous years as there was a new CF catheter launch in 2016 (Fig. 3). Catheter launches correlate with a spike in the AEF rate. For these reasons, a direct statistical comparison of these 2 years alone is inadequate. As such, this manuscript has focused on the comparison of the overall rate between 2014 and 2017 which better accounts for the change in usage and other associated variations due to chronology.

### Retrospective workflow analysis of AEF cases

To understand how ablation parameters from AEF cases compare with the recommendations of the 2017 HRS/EHRA/ECAS/APHRS/SOLAECE Consensus Statement, the clinical workflow on the LA posterior wall was investigated through analysis of seven anonymized (patient-deidentified) AF ablation CARTO® 3 System log files from CF procedures that resulted in AEF (Table 1 and Table 2). For each of these cases, a wide area circumferential ablation strategy had been employed. As noted in Sect. 2, these files were voluntarily provided when AEF occurred. In the absence of a control group, the explored parameters serve only as hypothesis generating data. Further studies will need to be carried out to prove these concepts.

**Table 1:** Patient and clinical characteristic information from retrospectively analyzed AEF cases

Country	Age	Sex	Weight (kg)	Outcome	PPD	Esophageal probe
USA	48	M	Unk	Stable	14	Yes
USA	Unk	F	Unk	Death	7	Yes
USA	72	F	63.5	Stable	6	Yes
UK	53	M	90	Death	16	No
UK	60	M	Unk	Stable	3	Unk
UK	68	F	Unk	Death	30	Unk
Belgium	65	M	Unk	Death	0	Yes

Due to legal and technical constraints, it is difficult to obtain procedure log files for retrospective analysis. Nevertheless, a limited number (7) of CARTO® 3 System log files from procedures involving CF catheters with a documented AEF were voluntarily provided by physicians. Physicians provided patient-blinded CARTO® 3 System log files from previously completed real-world cases where the patient experienced an AEF between 2014 and 2018. All patient information was deidentified

*PPD* post-procedure day

**Table 2:** LA posterior wall ablation parameters from retrospectively analyzed AEF cases

AEF case number		1	2	3	4	5	6	7	Mean ± SD
Power									
Total no. of VisiTags on posterior wall		50	52	64	50	20	58	30	46 ± 16
% of VisiTags > 25 W		82%	100%	80%	100%	100%	100%	100%	95 ± 9%
Maximum power on posterior wall		41 W	42 W	42 W	42 W	46 W	40 W	46 W	43 ± 2 W
Average maximum power on posterior wall		36 W	32 W	33 W	35 W	31 W	31 W	27 W	33 ± 5 W
Contact force									
Max. CF when > 25 W on posterior wall		51 g	57 g	49 g	34 g	52 g	31 g	32 g	47 ± 10 g
CF as % above 10 g on posterior wall		510%	570%	490%	340%	520%	310%	320%	470 ± 100%
Average CF on posterior wall		18.3 ± 9.1 g	13.4 ± 5.9 g	18.4 ± 10.5 g	17.3 ± 9.5 g	17.3 ± 11.9 g	17.2 ± 6 g	21.7± 11.3 g	17 ± 10 g
% of VisiTags with:	CF < 10 g	18%	15%	41%	32%	15%	29%	13%	23 ± 11%
	10 ≤ CF < 20 g	44%	52%	25%	30%	25%	57%	57%	41 ± 15%
	20 ≤ CF < 30 g	28%	21%	13%	24%	45%	12%	27%	24 ± 12%
	30 ≤ CF < 40 g	8%	8%	17%	14%	10%	2%	3%	9 ± 6%
	CF ≥ 40 g	2%	4%	5%	0%	5%	0%	0%	2 ± 2%
Duration									
Total posterior wall RF duration		15 min	12 min	8 min	9 min	5 min	10 min	6 min	9 ± 4 min
Max. tag duration on posterior wall		43 s	40 s	36 s	36 s	39 s	23 s	28 s	35 ± 7 s
% of VisiTags > 20 s on posterior wall		38%	15%	5%	14%	75%	9%	90%	35 ± 34%

The available clinical characterization information is summarized in Table 1. Table 2 summarizes the ablation parameters on the LA posterior wall. The average maximum contact force on the LA posterior wall in all 7 cases, was 47 ± 10 g. On average per case, 77 ± 10%, 35 ± 14%, and 11 ± 7% of posterior wall VisiTags recorded maximum contact force above 10 g, 20 g, and 30 g, respectively. Most ablations (95 ± 9%) on the LA posterior wall exceeded 25 W (average maximum power 43 ± 2 W) with the maximum power being above 40 W in all 7 cases. There were multiple ablations (35 ± 34%) longer than 20 s in each of these 7 patients. Total posterior wall ablation lasted 9 ± 4 min (Table 2).

Note, this investigation was not able to confirm if these type of ablation parameters were used without injury in cases in which no AEF was generated or in non-CF device cases where AEFs did occur.

## Discussion

The goal of this study was to compare the true rates of AEF between CF-sensing and non-CF-sensing catheters. A second goal of this paper was to assess the workflow parameters that were employed in seven patients who developed an AEF. Recent studies have relied on the absolute number of AEF events from the MAUDE database to estimate the rates of AEF [8]. In contrast, this analysis provides the closest estimate of the true rate of AEF, since the number of reports of AEF was normalized to catheter sales data (a surrogate for usage volume).

There are several important findings of this study. First, there was no difference in the rate of AEF with CF and non-CF ablation catheters between 2014 and 2017 as a whole. Second, the rate of AEFs with either catheter type was extremely low (0.005% versus 0.006%, respectively) [4]. And finally, we provide examination of posterior wall ablation parameters from seven patients that experienced an AEF with a CF-sensing catheter, the most detailed observational analysis of the ablation parameters on the posterior wall to date, which shows contact force, and power measurements that are above those recommended by the 2017 HRS/EHRA/ECAS/APHRS/SOLAECE Consensus Statement [4]. This unique information is of value to electrophysiologists who perform AF ablation procedures.

To our knowledge, this is the first report where the incidence of AEF was expressed as a function of device usage from post-market safety surveillance data. This approach not only provides the most accurate to date estimation of AEF incidence but also allows for comparison between different catheter types. We normalized the number of AEF reports from a device database to device sales, as a proxy for number of procedures, and found a very low incidence rate for both CF and non-CF catheters compared to other published literature [4]. The difference between our results and other reported AEF rates may be due to various factors. First, this may be because we only analyzed events that were reported with Biosense Webster catheters. Second, due to the extreme rarity of AEF, it is hard for any other study to accumulate enough cases for comprehensive analysis and thus numbers can be skewed. Lastly, and most importantly, until now, no study could assess the true rate of the AEF, since they did not have the number of procedures performed. Here, we used the sales numbers as the closest approximation.

A recent study used the publicly searchable MAUDE database and found that AEF comprised 5.4% of CF-related reports. Moreover, the authors stated a significantly lower number of non-CF-related AEF reports (0.9%) and concluded that CF ablation catheters cause more AEFs than non-CF catheters [8]. The main limitation of this approach is that it only considers the proportion of AEF among the reports filed in the MAUDE database and does not account for a much greater usage of CF catheters in LA procedures. In other words, the numerator is the number of CF-related reports in the MAUDE database, but the denominator is missing. Therefore, this report could not provide information on the incidence of AEF with different catheter types. Moreover, if the clear majority of AFib procedures (with higher chance of AEF) are performed using CF technology, it is not surprising that there are more CF- than non-CF-related AEF reports in the MAUDE database. In the present study, we addressed this limitation by using the device sales as the denominator and show that, despite more frequent usage in LA procedures, CF ablation catheters have extremely low rates of AEFs, which is statistically not different from non-CF catheters (*p* = 0.69).

Analysis of temporal trends of AEF incidences showed a robust downtrend for both CF and non-CF devices, with the highest incidence in 2014, when THERMOCOOL SMARTTOUCH® CF Catheter technology was launched broadly in the USA. The fact that the incidence of AEF was high for both CF and non-CF catheters, suggests that it was workflow-driven rather than directly related to CF technology. Interestingly, the highest rates of AEF are seen when new catheters are introduced to the market, likely suggesting that there is a learning curve. Our proposal that a learning curve may contribute to these spikes is complimented by literature which has shown that overall adverse event rates are highest for less experienced physicians and low-volume hospital sites [9, 10].

Regardless of the type of catheter used, delivering unintentional high CF with non-CF-sensing catheters, or intentionally high CF while using CF-sensing devices, will increase the patient’s risk of AEF [3, 11, 12]. For example, higher contact force, together with higher power and longer duration, has been shown to result in deeper lesions, more tissue heating and steam pops—all factors that if not managed proactively may lead to AEF and other complications [6, 13]. Further, the association of higher-CF leading to adverse event (cardiac tamponade) was discussed in the SMART-AF study [3]. Although the sample is limited, analysis of workflow data from a collection of seven AEF events between 2014 and 2018 showed an average maximum contact force of 47 ± 10 g, average maximum power of 33 ± 5 W, and multiple RF ablation sessions longer than 20 s on the posterior wall. The advantage of CF ablation catheters is that physicians receive real-time force feedback, which in some cases has been shown to reduce injury [12, 14]. However, it is important that physicians continue to use their best clinical judgment in their clinical practice to ensure patient safety by lowering the values of the parameters.

## Conclusions

CF and non-CF ablation catheters were found to have very low and similar rates of AEF between 2014 and 2017. Because CF catheters are used two-to-five times more frequently in left-sided procedures than non-CF catheters, AEF rates would have been expected to be higher with CF vs. non-CF catheters. The similar rates provide further evidence of the safety of CF technology. In retrospective analysis of AEF cases, high force, high power, and long duration of RF were observed on the posterior wall of the LA, although no causality for the AEFs could be definitively determined. Additional investigation is required to better understand the most efficient ways to maximize the efficacy of AF ablation while mitigating AEF risk.

## Limitations

There are several limitations that need to be considered when reading this paper. First, we only provide the relative rates of AEF normalized to catheter sales rather than the actual number of reports of AEF. This approach was employed because the precise sales information is proprietary and is not able to be publicly released. We do not believe this is a significant limitation to this analysis as what is most important is the rate of AEF rather than the number of AEFs. A second limitation is that our analysis is not limited to AF ablation procedures but includes all types of ablation procedures in both the left and right atrium. For a 3-year period (2015–2017), we were able to track what proportion of CF catheter usage was in the left atrium presumably for AF ablation. This analysis revealed that 85% of ablation procedures in the LA were performed with CF catheters by the end of 2017. This important observation would accentuate any possible increased risk of AEF associated with CF catheters. But this is not what was seen.

It is important to note that this study may be impacted also by a potential underreporting bias of AEF numbers. As AEFs typically occur several days to weeks after an ablation procedure, and not all EP centers have a rigorous follow-up of atrial fibrillation patients, there is some potential for underreporting. However, any possible under-reporting bias should affect all adverse event reporting databases similarly. In fact, prior studies that were based on the MAUDE database for cardiovascular devices conclude there is systemic underreporting of adverse events [15]. Nonetheless, CF and non-CF technologies are similar devices, and there should be no expected difference in the propensity for reporting between them.

Lastly, the number of reports of AEF was normalized to catheter sales data (a surrogate for usage volume). Not knowing the exact number of procedures/patients is a limitation of post-market surveillance data. However, the denominator presented here creates the best ability to approximate a true rate of AEF.
